# Small but troublesome: accessory ossicles with clinical significance

**DOI:** 10.1590/0100-3984.2017.0147

**Published:** 2018

**Authors:** André Vaz, Cesar Rodrigo Trippia

**Affiliations:** 1 MD, Resident in Radiology and Diagnostic Imaging at the Hospital Nossa Senhora das Graças, Curitiba, PR, Brazil.; 2 MD, Radiologist, Preceptor of the Radiology and Diagnostic Imaging Residency Program of the Hospital Nossa Senhora das Graças, Curitiba, PR, Brazil.

**Keywords:** Bone and bones, Anatomic variation, Magnetic resonance imaging, Tomography, X-ray computed, Radiology

## Abstract

Accessory ossicles are supernumerary and inconstant structures that are not caused by fractures. Derived from unfused ossification centers, accessory ossicles were first described by Vesalius in 1543. For centuries, they were believed to be asymptomatic. However, with advances in radiology techniques, many have been associated with painful syndromes. Although the original descriptions date from the sixteenth century, the subject is little discussed and, in some cases, controversial. The objective of this study was to describe the radiological aspects of a series of accessory ossicles and to review the evolution of their various descriptions, in order to revive discussion of the subject.

## INTRODUCTION

Accessory ossicles are inconstant, independent, and considered well formed bones, not arising from fractures or other diseases, although they are equally susceptible to both conditions^(1)^. They are derived from unfused ossification centers^(2)^, which can form free ossicles, sesamoid ossicles (imbedded in a tendon), or bipartite ossicles (congenital non-traumatic division)^(1)^. Although accessory ossicles were first described by Vesalius in 1543^(1)^, the subject attracted little attention until recently, because, prior to the advent of radiology, there was a lack of knowledge with respect to their clinical implications. There is now greater interest in the area, due to reports of pain syndromes related to these ossicles^(2)^.

The purpose of this study was to highlight a series of cases of accessory or bipartite ossicles that were of clinical importance, either because they provoked symptoms or because their differential diagnoses were important. Data related to the cases were obtained from the archives of one of the authors.

## OS VESALIANUM PEDIS

**Figure 1:**
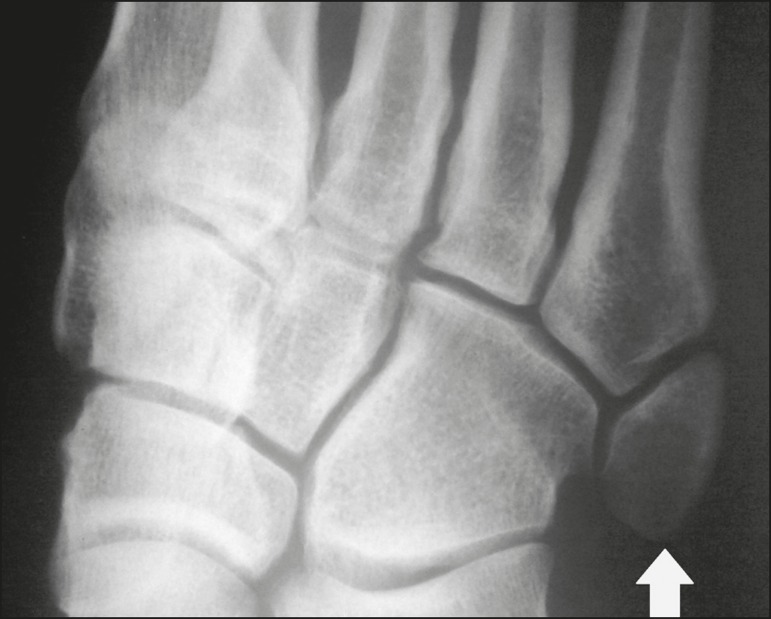
X-ray of the left foot (detail), in an oblique view, showing an unfused ossicle at the base of the fifth metatarsal (arrow), with well-formed cortical margins and apparent articulation with the base of the fifth metatarsal and cuboid, separated by a radiolucent line of uniform thickness, characteristic of os vesalianum pedis.

The accessory ossicle adjacent to the base of the fifth metatarsal is known as os vesalianum pedis (Figure 1). It was described by Pfitzner in 1900 as an ossicle that would constitute the tuberosity of the fifth metatarsal, however that definition is contested because some consider it to be adjacent to a normally developed tuberosity^(1,2)^.

The incidence of the os vesalianum pedis ranges from 0.1% to 0.4%^(2,3)^. Although it is typically considered an asymptomatic incidental finding, there have been reports of pain syndromes^(4)^.

The os vesalianum pedis should be distinguished from the os peroneum, the apophysis of the fifth metatarsal, Iselin's disease, avulsion fracture of the tuberosity, Jones and stress fracture of the fifth metatarsal. Identification of a joint with the cuboid can guide the diagnosis, because it suggests os vesalianum pedis^(5)^, although computed tomography (CT) or magnetic resonance imaging (MRI) might be necessary for the accurate diagnosis of the finding.

The os peroneum is a sesamoid bone within the peroneus longus tendon, adjacent to the calcaneocuboid joint (more proximal than the os vesalianum pedis), and is relatively common, with an incidence of 9-26%^(2,3,6)^.

The apophysis of the fifth metatarsal is parallel to the long axis of the metatarsal diaphysis^(2,3)^ and can be seen on X-rays in individuals 9-11 years of age, before it fuses with the bone axis at 12-16 years of age^(3)^.

Iselin's disease consists of self-limiting apophysitis caused by traction of the base of the fifth metatarsal, resulting from repeated microtrauma. An X-ray of the foot can show enlargement or fragmentation of the osteochondral joint, and MRI can show bone edema^(7)^.

Avulsion fracture of the tuberosity of the fifth metatarsal results from forced inversion of the foot. The increased tension on the peroneus brevis tendon in the movement causes apophysis detachment and the trace of the fracture is transverse at the base of the fifth metatarsal^(2)^.

The Jones fracture was described in 1902, in a report of six cases (including that of the author), as a fracture transverse to the long axis of the fifth metatarsal approximately 2 cm from its base, between the insertions of the peroneus brevis and peroneus tertius tendons^(8)^, all of the cases being attributed to forced inversion and plantar flexion of the foot^(9)^.

A stress fracture evolves from microfractures to complete fracture, on average, 1.5 cm from the proximal diaphysis of the fifth metatarsal^(8)^. Poor irrigation of the metadiaphyseal region usually impairs the healing of these fractures, which can delay or prevent the consolidation of the fragments^(8)^. Therefore, the sequelae of such fractures can be confused with a diagnosis with os vesalianum pedis.

## BIPARTITE SCAPHOID

**Figure 2:**
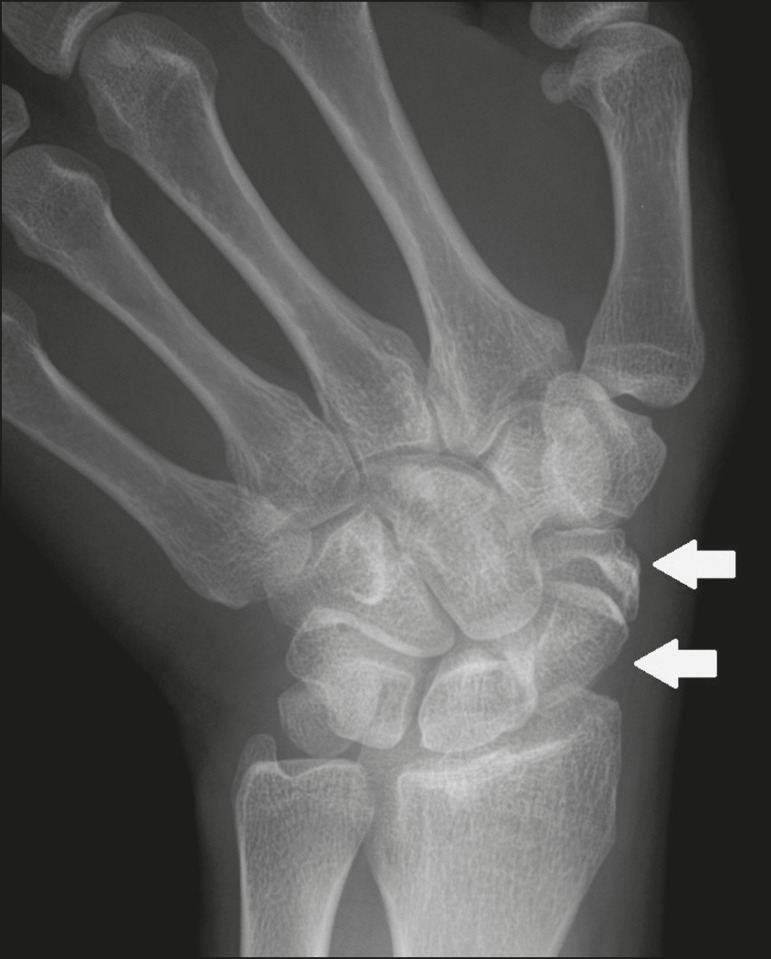
X-ray of the right wrist in ulnar deviation showing a divided scaphoid (arrows) in the distal third. Note that the medial fragment is larger than the lateral fragment, both having well-formed, regular margins suggestive of bipartite scaphoid.

The earliest reports of divided scaphoid were published in 1877 and 1895 by Gruber and Pfitzner. Although the authors dissected 3007 and 1450 specimens, they identified a divided scaphoid in only 4 (0.13%) and 9 (0.62%) of the cases, respectively. Fracture was the main hypothesis to explain the division, although a hypothesis of non-fusion of the radial and ulnar ossification centers of the scaphoid, forming a bipartite scaphoid (Figure 2), has also been proposed^(10)^.

In 1906, Dwight reported a case of congenital bipartite scaphoid and was able to confirm the non-fusion hypothesis^(11)^. However, that theory has been questioned. Louis et al.^(10)^ reviewed 17,439 wrist X-rays (5365 were in children between 54 and 150 months of age) and found multiple scaphoid ossification centers in three cases (~0.01%), all in children. Despite the lack of follow-up of those three cases, the authors concluded that the putative multiple ossification centers likely coalesce to form a single scaphoid in adults.

Recent evidence supporting the non-fusion hypothesis was published in 1990, when Doman et al.^(12)^ reported the case of a young girl with two scaphoid ossification centers, one on each side, and subsequent evolution (between 8 and 17 years of age) to symptomatic bilateral bipartite scaphoid. The differential diagnosis would be a scaphoid fracture, although that is uncommon in children and, when it occurs, the division of the fragments is typically in the distal third. The authors concluded that, although extremely uncommon, one of the causes of division could be congenital unfused ossification centers (bipartite scaphoid).

The diagnostic criteria described by Bunnell are currently accepted: absence of a history of trauma; presence of bilateral scaphoid division; equal size and density of both scaphoid ossicles; the absence of degenerative changes in both scaphoid components or another site in the wrist; and rounded, regular margins of both scaphoid components^(13)^. Although one of the pillars of diagnosis is the absence of degenerative changes, there have been reports of cases of symptomatic bipartite scaphoid with osteoarthritis in its components^(12,14,15)^. Therefore, the diagnosis of a divided scaphoid can be challenging, especially when the patient is symptomatic, because it could represent a bipartite scaphoid with degenerative changes or a fractured scaphoid with pseudoarthrosis.

## OS TRIGONUM

**Figure 3:**
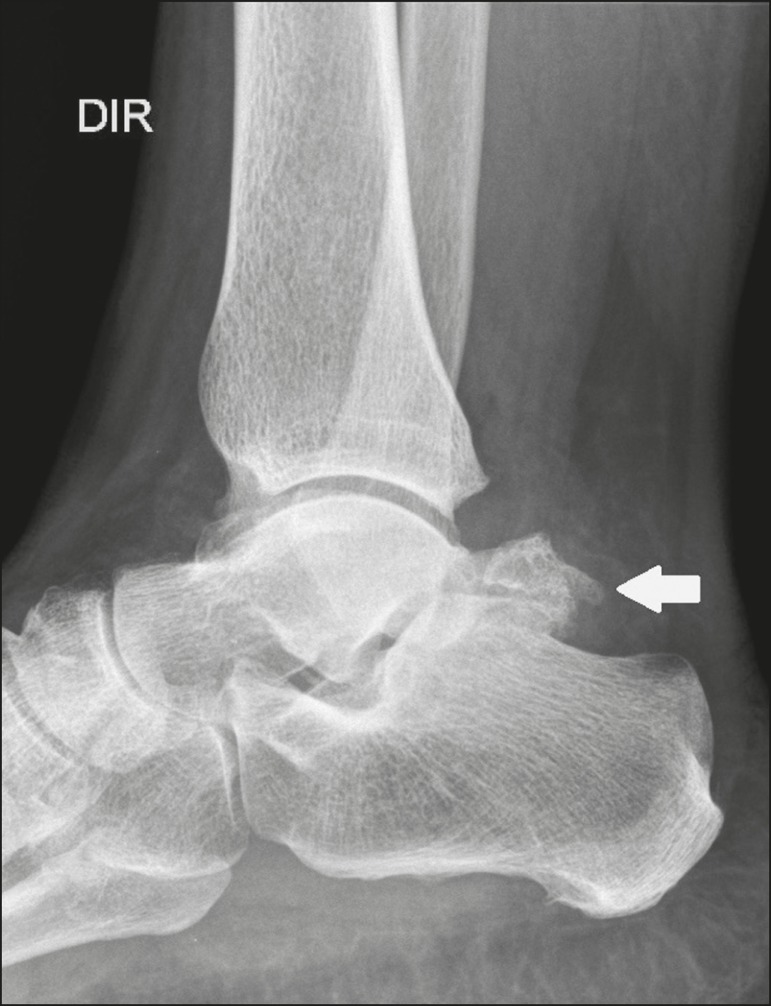
Lateral X-ray of the right ankle showing a triangular ossicle (arrow), with an irregular surface, posterior to the talus, the joint space between the ossicle and the talus not being clearly defined. On X-ray, the differential diagnosis includes Stieda's process and os trigonum.

**Figure 4:**
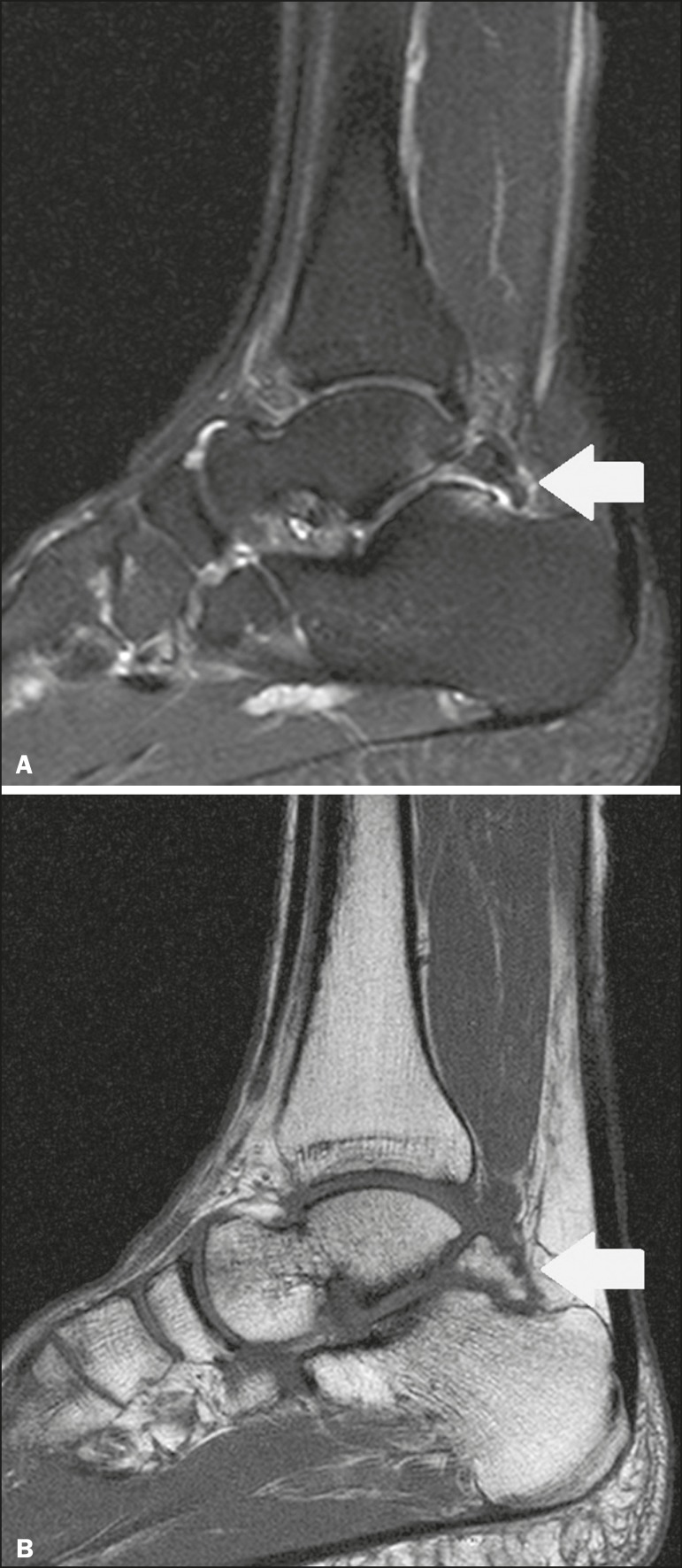
Sagittal T1-weighted and T2-weighted MRI scans (**A** and **B**, respectively) clarify the diagnosis of the case depicted in Figure 3, because they reveal a joint between the ossicle and the talus, consistent with os trigonum. In addition to the joint, note the bone edema, which is suggestive of inflammatory changes.

The os trigonum (Figures 3 and 4) is an accessory ossicle posterior to the talus and is relatively common, with an incidence of 7-8%^(16,17)^. The posterior process of the talus contains two tuberosities, the medial and the lateral, divided by the sulcus of the flexor hallucis longus tendon^(18)^. The os trigonum articulates, via a synchondrosis, with one of the tuberosities of the posterior process of the talus, can articulate with the calcaneus, and rarely, can occur in duplicate, one os trigonum being seen in each tuberosity^(1,17,19)^. It was first described by Rosenmüller in 1804^(17)^, being classified as an accessory ossicle by Gruber in 1864 and Stieda in 1869. However, in 1882, Shepherd concluded that it was the result of fracture of the posterior process of the talus^(20)^. Although Shepherd's error was corrected in that same year by Turner^(21)^, the incorrect term "Shepherd's fracture" can still be found in the literature^(1)^.

The same ossification center that forms the os trigonum, when merged with the posterior process of the talus, can create a more prominent tuberosity known as Stieda's process^(22)^. Both bony appendages may cause talar compression syndrome or posterior ankle impingement syndrome, especially in individuals who perform repeated plantar flexion of the foot^(22,23)^. This syndrome occurs in dancers and soccer players, because, by maintaining the *pointe* and *demi-pointe* ballet positions^(23)^ or by kicking the ball^(24)^, they compress the adjacent structures^(23)^, such as the posterior talar, intermalleolar, and tibiofibular ligaments or the flexor hallucis longus tendon itself^(22)^.

The posterior ankle impingement syndrome includes pain in the posterolateral aspect of the ankle that worsens upon vigorous plantar flexion. Lateral ankle X-rays can show the os trigonum and Stieda's process, and an additional incidence in plantar flexion may be useful to identify the impact of the bony protuberance on the tibia^(24)^; however, soft tissues are better studied on MRI^(22)^. Excision of the os trigonum or Stieda's process is usually sufficient to reduce pain^(23)^.

The differential diagnosis of talar compression syndrome includes tenosynovitis or rupture of the flexor hallucis longus tendon, tenosynovitis of the peroneus tendons in the lateral portion of the ankle, and tendonitis of the calcaneal tendon^(23)^. Given the incidence of findings in the tuberosities of the posterior process of the talus, talar compression syndrome and posterior ankle impingement syndrome should be considered in patients with ankle pain.

## OS ACROMIALE

**Figure 5:**
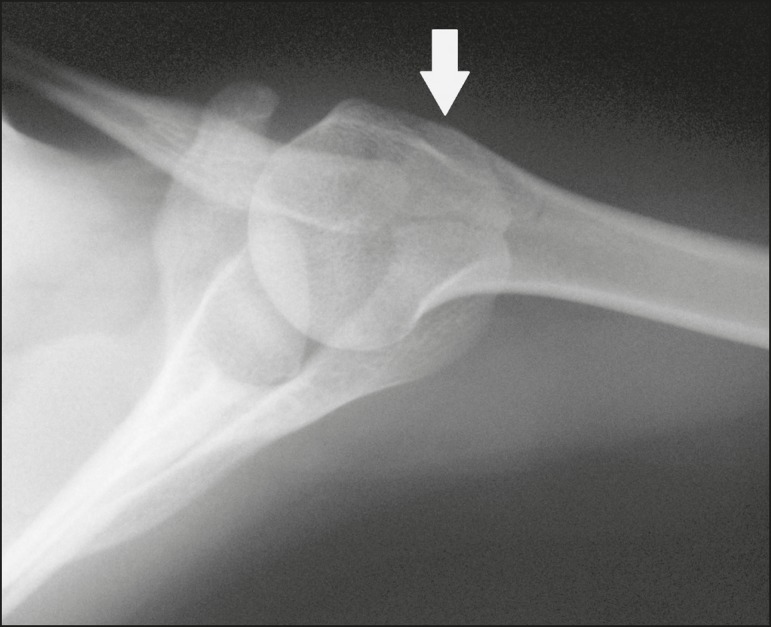
Axillary X-ray view showing a triangular ossicle distal to the acromion (arrow), separated by a line of uniform thickness, consistent with os acromiale.

**Figure 6:**
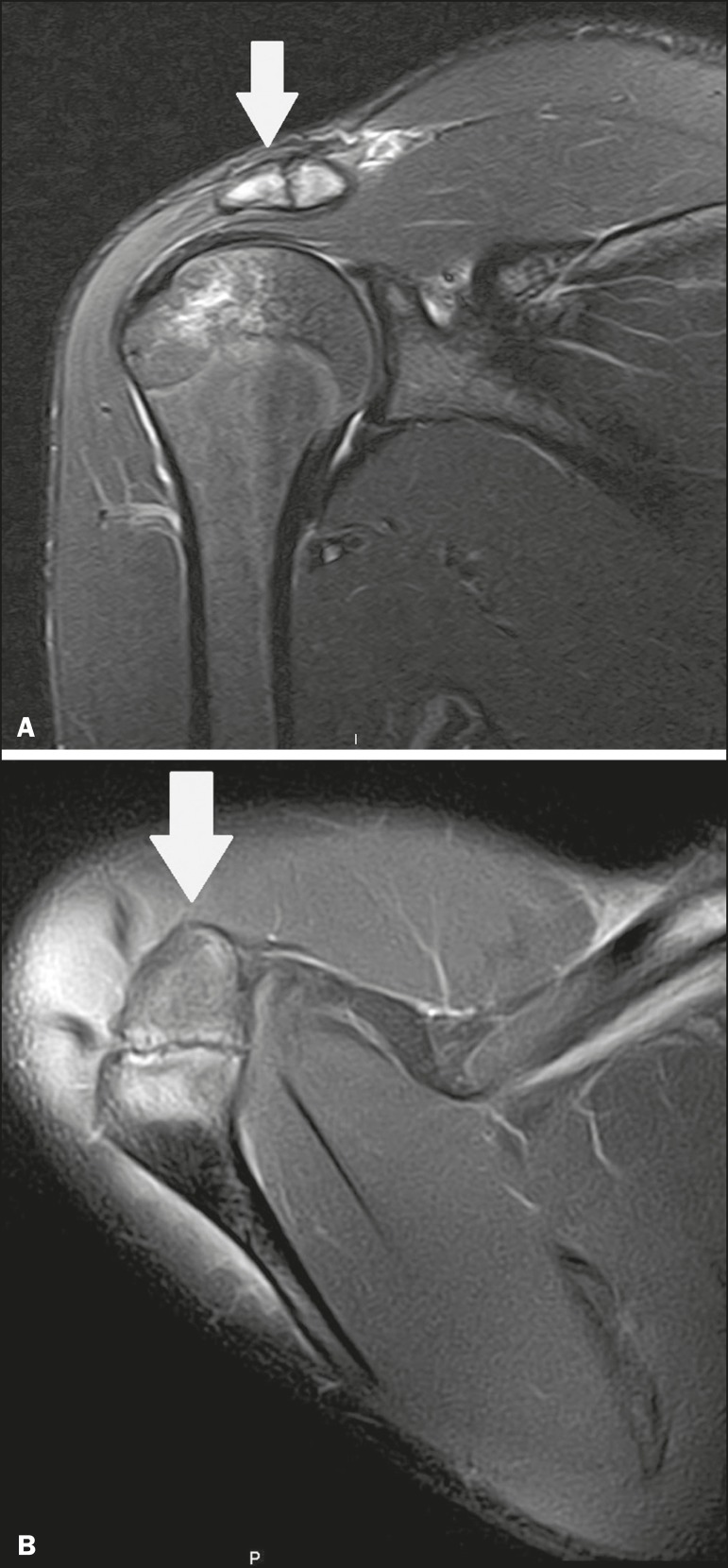
T2-weighted axial and coronal MRI scans of the right shoulder (**A** and **B**, respectively) showing bone edema, suggestive of inflammatory changes, in the os acromiale and in the distal portion of the acromion.

Os acromiale (Figures 5 and 6) is an accessory ossicle resulting from failure to fuse of one of the four epiphyses of the acromion^(25)^. The divided acromion was first described by the Roman surgeon Galeno between the second and third centuries^(26)^, and in 1863 Gruber described 3 cases among 100 dissected cadavers, attributing the division to unfused epiphyses and referred to the accessory ossicle as os acromiale^(26,27)^.

Despite the results obtained by Gruber, Struthers et al., in 1896, dissected 14 cadavers and attributed the division of the acromion to fractures that, with the constant movement of the scapula against the clavicle, did not consolidate and formed a joint between the two portions of the acromion^(28)^. Shortly thereafter, anatomists, such as Gray and Cunningham, adopted the hypothesis of unfused epiphyses but did not rule out the hypothesis of fractures in some cases^(27)^.

To mitigate the discussion and end the divergence, Liberson, in 1937, reported 25 cases of os acromiale identified on shoulder X-rays and proposed three criteria to distinguish between those resulting from fracture and those representing os acromiale^(25)^: bilaterality; rounded borders and constant thickness of the cleavage line between the ossicle and the remainder of the uniform acromion; and position of the ossicle at or higher than the rest of the acromion.

Neer, in 1972, reported 50 cases of shoulder impingement syndrome that underwent acromioplasty. In some cases, os acromiale was identified during surgery and was implicated as one of the causes of rotator cuff rupture^(29)^. Similar findings from a series of eight cases by Mudge et al. in 1984 confirmed the hypothesis of rupture of the rotator cuff related to os acromiale^(30)^.

Although os acromiale can be identified on axillary X-rays, CT or MRI can be necessary in order to visualize degenerative changes of the ossicle or indications of rotator cuff injury^(26,31)^, such as edema or calcification in the supraspinatus tendon. Osteophytosis of the margins of os acromiale indicates instability that can result in impingement syndrome^(32)^.

The identification of os acromiale is crucial in the context of shoulder impingement syndrome, because the presence of the accessory ossicle can alter the surgical approach^(29)^.

## OS HAMULI PROPRIUM

**Figure 7:**
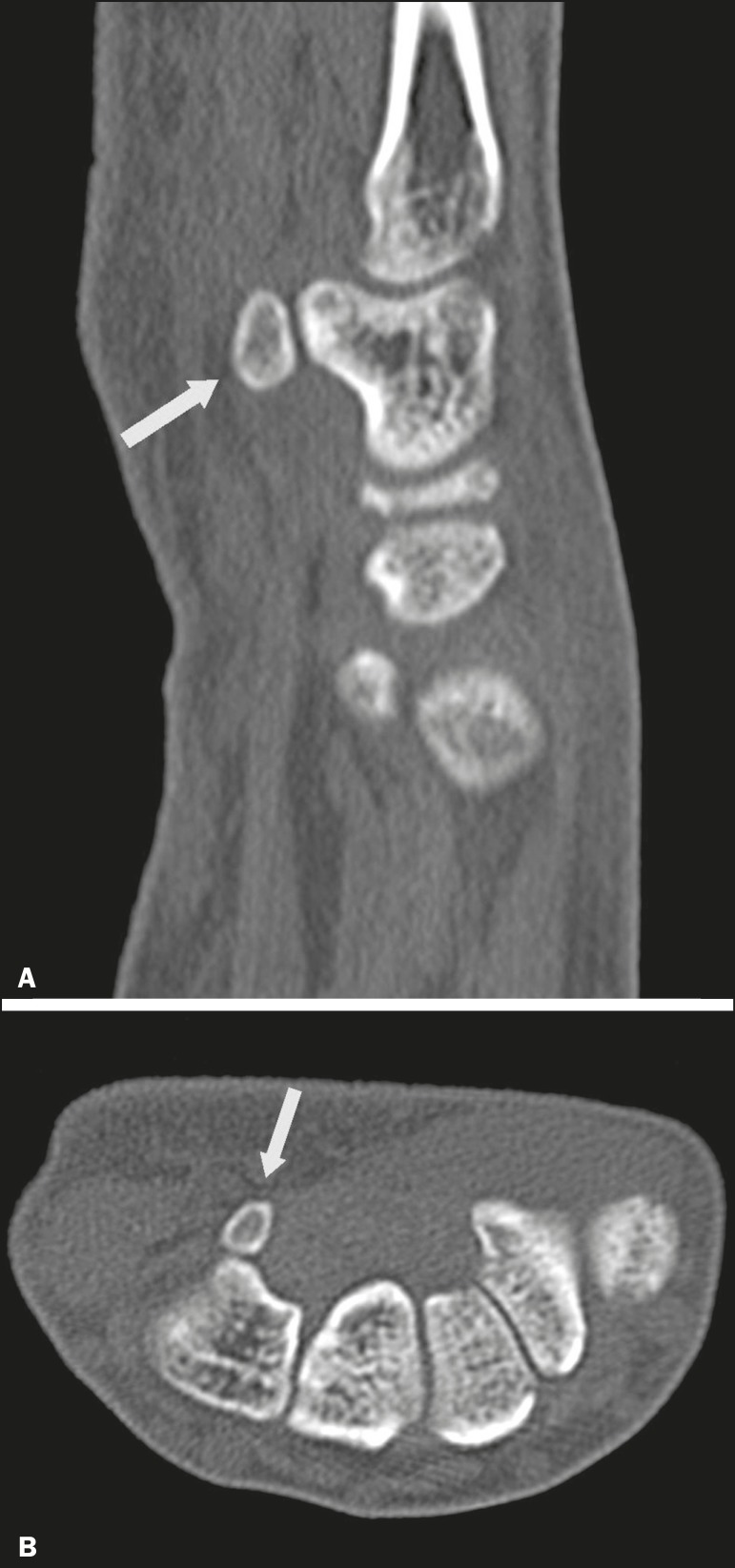
Axial and sagittal CT scans of the wrist (**A** and **B**, respectively) demonstrating, at the level of the hamate, a separation from its hamulus, with rounded and regular contours, suggestive of os hamuli proprium (arrows).

The os hamuli proprium, or bipartite hamulus (Figure 7), is a rare accessory ossicle arising from failure of the hamulus ossification center to fuse. It was first reported by Thelineus in 1896^(33)^. In 1932, Bugart evaluated 1452 wrist X-rays and identified an os hamuli proprium in 1 (0.06%), whereas Chow et al. identified it in 42 (1.3%) of 3218 wrist X-rays evaluated between 1989 and 2002^(33,34)^.

The hook of the hamate is an important landmark in the wrist, because it is the lateral and distal delimiter of Guyon's canal, which contains the fat and ulnar artery, nerve, and veins. Its other delimiters are the pisiform bone (proximally and medially); the volar carpal ligament (anteriorly); and the pisohamate and transcarpal ligaments (posteriorly). The hamulus serves as a pulley for the flexor pollicis profundus tendon and insertion site of the ulnar border of the transverse carpal ligament, the pisohamate ligament, and the flexor digiti minimi brevis and opponens pollicis muscles. Hamate hook fracture can occur in athletes engaged in sports that require palmar gripping of a racket, club, or bat (tennis, golf, or baseball), as well as in cyclists and jackhammer operators^(33,35)^. The tensile forces that result in a fractured hamate complicate its fusion and favor pseudoarthrosis^(33)^. Therefore, an unfused fracture of the hamulus is the main differential diagnosis of os hamuli proprium.

Pain and paresthesia in the hypothenar eminence with irradiation to the fourth and fifth fingers may be caused by compression of the ulnar nerve at the level of Guyon's canal. This clinical entity was designated ulnar tunnel syndrome by Dupont et al., in 1965^(36)^. Neuropathy of the ulnar nerve was thought to be associated with hamate hook fracture, until 1981, when Greene et al. reported a case of bipartite hamulus associated with ulnar tunnel syndrome^(33)^. The presence of os hamuli proprium might also be associated with carpal tunnel syndrome, given that Chow et al., in 2005, found that 95.2% of individuals with bipartite hamulus also had carpal tunnel syndrome^(34)^.

The differential diagnosis of pain in the hypothenar eminence includes the following: fracture or dislocation of the pisiform bone; pyramidal osteochondral fracture; hamate hook fracture; carpal ulnar flexor tendonitis; tenosynovitis of the flexor pollicis tendon; osteoarthritis of the piso-pyramidal joint; ulnar tunnel syndrome; hypothenar hammer syndrome; hand-arm vibration syndrome; ganglion cyst; schwannoma; hemangioma; lipoma; and osteoid osteoma^(35)^. A carpal tunnel view X-ray can identify separation of the hamulus from the hamate, although CT might be indicated to distinguish between fracture and bipartite hamulus, which will present a well-defined regular margin^(33)^, and MRI might be indicated to assess the involvement of the ulnar nerve^(35)^.

Pain in the hypothenar eminence is a common complaint and has a broad differential diagnosis^(35)^. Investigation can begin with X-rays, which can reveal, for example, os hamuli proprium, although other methods might be needed to clarify the diagnosis.

## TYPE 2 ACCESSORY NAVICULAR BONE

The navicular bone can present three different alterations that are considered accessory: the first, described by Bauhin in 1605, was designated os tibiale externum^(17)^ and was found to correspond to a sesamoid in the posterior tibial tendon, which inserts into the posterior portion of the navicular tubercle. Von Luschka, in 1858, reported a variation of Bauhin's description in a 17-year-old individual. The adolescent presented an ossicle in the posterior region of the navicular tubercle, albeit articulated and surrounded by a joint capsule^(37)^. The third alteration was described in 1914 by Geist, who found 14 feet with accessory navicular bones among 100 X-rays. Among these 14 feet, the authors found some (the exact number was not reported) in which the ossicle had fused to the navicular bone, forming a more prominent tuberosity^(17)^.

**Figure 8:**
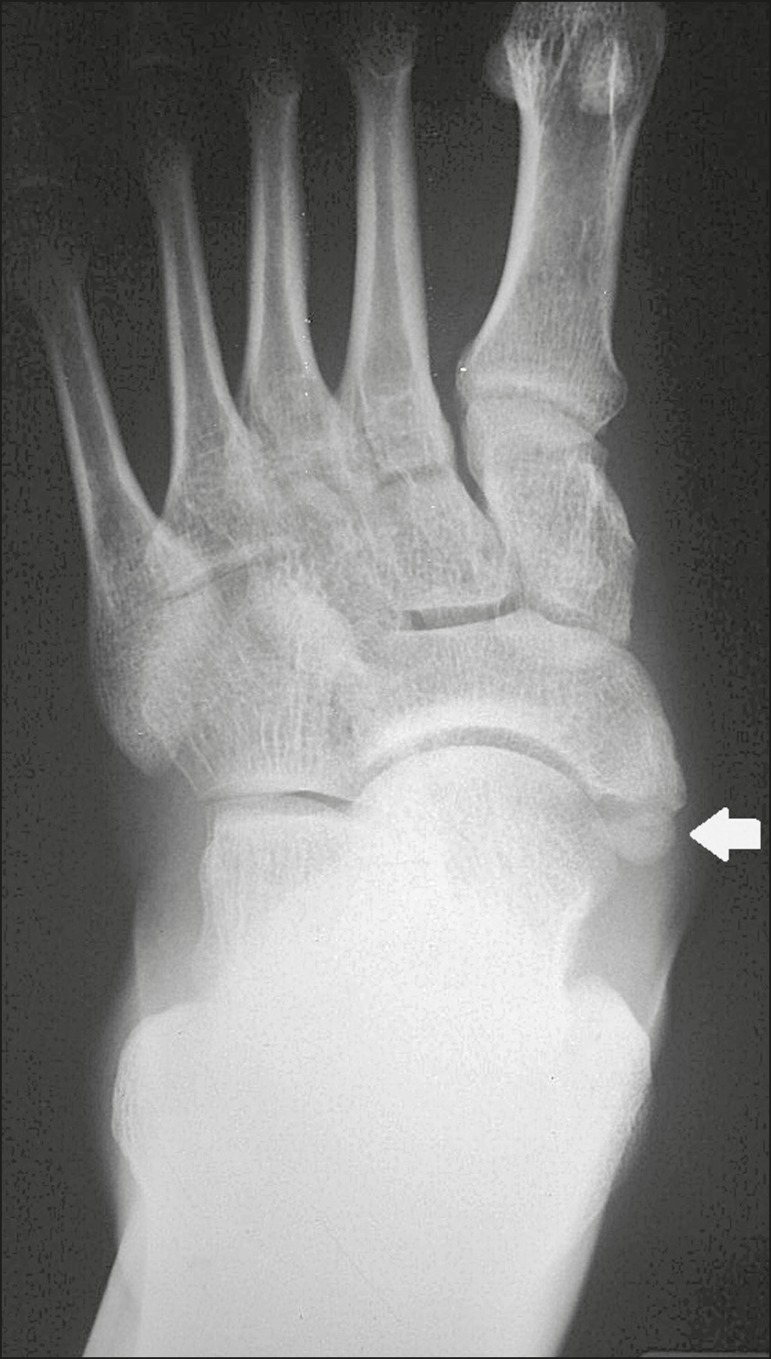
Anteroposterior X-ray of the left foot showing a triangular ossicle posteromedial to the navicular bone (arrow), with regular margins and apparent articulation with the navicular bone, consistent with a type 2 navicular accessory bone.

**Figure 9:**
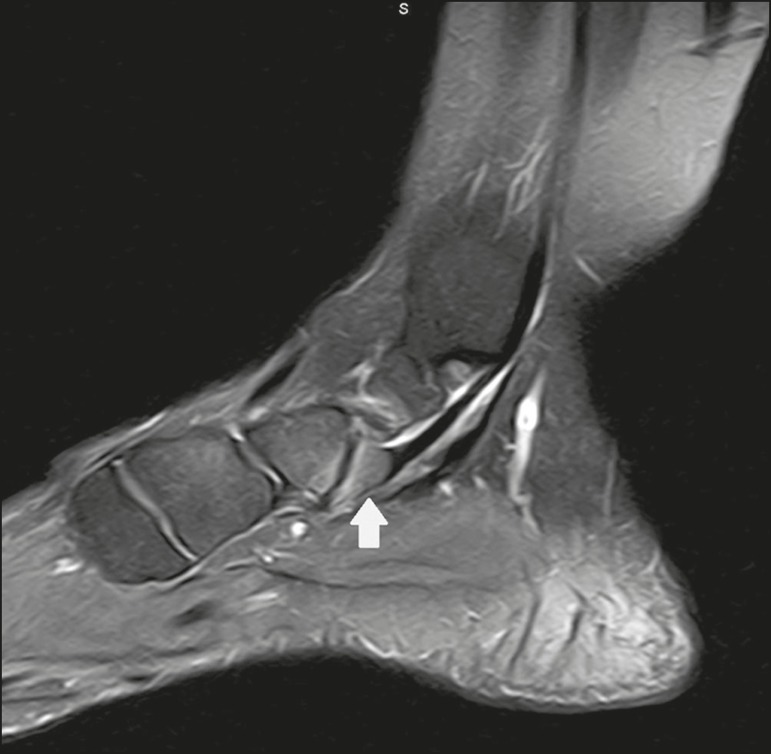
Axial T2-weighted MRI scan of the foot showing the insertion of the posterior tibial tendon and inflammatory changes in the accessory navicular bone (arrow).

The accessory navicular bone is currently classified as follows^(38)^: type 1, when rounded, measuring between 2 and 6 mm and located inside the posterior tibial tendon (corresponds to the description of os tibiale externum and has been observed in 30% of cases); type 2 (Figures 8 and 9), when triangular, articulated by a fibrocartilaginous joint to the navicular bone and measuring approximately 12 × 9 mm (corresponds to von Luschka's description and has been observed in 70% of cases); and type 3, when the navicular bone has a corniced appearance (a prominent tubercle that corresponds to the fusion described by Geist).

In 1978, Veitch reported a series of 21 patients with pain in the medial aspect of the foot. Among the symptomatic feet, 91% had a type 2 accessory navicular bone and the remaining symptomatic feet had a type 3 accessory navicular bone. Several other asymptomatic feet (number not reported) had a type 1 accessory navicular bone.^(39)^. Other authors have found the same association between symptoms and a type 2 accessory navicular bone, observing that this type can evolve to osteonecrosis in symptomatic patients^(33,40)^.

An accessory navicular bone can be identified and classified on foot X-rays, although MRI can be necessary in symptomatic cases to identify signs of bone edema and changes in the adjacent soft tissue, such as the synchondrosis and of the posterior tibial tendon^(40,41)^.

## TYPE I BIPARTITE PATELLA

The patella is considered a sesamoid that works like a pulley, facilitating the extension of the knee^(42)^. A divided patella at the superolateral angle was first reported in 1883 by Gruber, who designated the condition bipartite patella. Other cases were reported and the division was attributed to fracture, until 1921, when Todd and McCally suggested that the patella could originate from more than one ossification center and that a divided patella could be caused by an anomaly of fusion of those centers^(43)^.

In 1935, George^(43)^ reported a case of bilateral divided patella identified during the autopsy of a 63-year-old man. Upon histological analysis of the fragments of the patella, the patellar capsule and the cartilage between the fragments were found to be intact, with no evidence of the fibrosis that would have suggested a previous fracture. On the basis of that finding, the author proposed that the acquired cause (fracture) and the congenital cause could be differentiated through histological analysis of the fragments. In a fractured patella, there is discontinuity of the patellar capsule and cartilage between the fragments, whereas both are intact in a bipartite patella.

**Figure 10:**
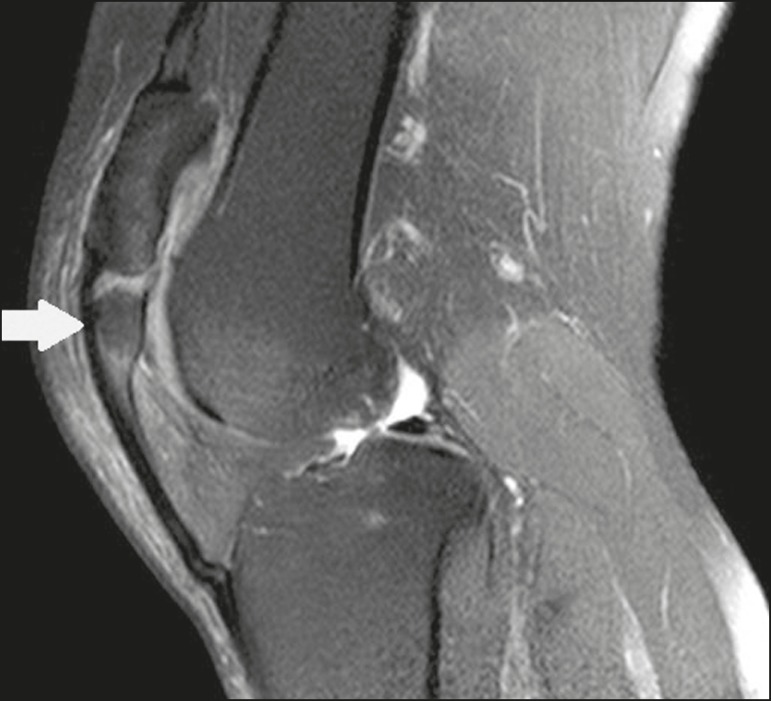
Sagittal T2-weighted MRI scan of the knee showing a bone fragment in the lower portion of the patella (arrow), next to the patellar tendon insertion, containing a discrete signal increase suggestive of inflammatory activity, consistent with either type I bipartite patella or Sinding-Larsen-Johansson disease.

A bipartite patella occurs in 2-3% of the population, is more common in men (at a ratio of 9:1), and is bilateral in approximately 50% of cases^(44,45)^. In 1943, Saupe proposed a classification of types of bipartite patella according to the location of the accessory fragment: type I (Figure 10), when it is in the lower pole of the patella (5% of cases); type II, when it is lateral (20% of cases); and type III, when it is superolateral (75% of cases)^(44)^.

The bipartite patella was believed to be an asymptomatic anatomical variation until 1977, when Weaver, reported 21 symptomatic cases, all with Saupe type III bipartite patella. The majority of cases were young athletes with knee pain during or after intense exercise, with no other probable cause and with improvement of symptoms after excision of the accessory fragment. The cause of the pain was attributed to abnormal mobility of the synchondrosis between fragments found during surgery^(44)^.

An accessory fragment can usually be identified on X-rays, in anteroposterior and axial patellar views, or on CT; however, MRI is necessary in order to clarify the diagnosis, because it can show edema in the accessory fragment and in the margins of the synchondrosis, between the fragments, in symptomatic patients^(45)^.

Despite the fact that the congenital ossification center non-fusion hypothesis is well established in the literature, there are reports of putative bipartite patellae in adults with knee pain in whom previous X-rays had shown normal patellae. According to Lawson, that suggests that the accessory fragment responsible for pain actually represents chronic chondro-osseous disruption rather than the degenerative changes of a congenital synchondrosis^(46,47)^. This pathophysiology resembles the findings of Osgood-Schlatter disease-traction injury in the tibial tuberosity at the patellar tendon insertion, which can cause fragmentation of the affected tibial portion-and Sinding-Larsen-Johansson disease-in the lower pole of the patella, at the level of the patellar tendon insertion, which can cause fragmentation of the affected patellar portion, a differential diagnosis for type I bipartite patella^(46-48)^. Therefore, the cause of bipartite patella has not yet been fully clarified.

The differential diagnosis of symptomatic bipartite patella includes the following^(48)^: Sinding-Larsen-Johansson disease, Osgood-Schlatter disease, jumper's knee (patellar tendonitis), and patella sleeve fracture (avulsion fracture of the lower patella pole).

The majority of reported cases of symptomatic bipartite patella occur in Saupe types II and III. The only reports of symptomatic type I bipartite patella are the four cases described by Okuno et al., in which there was traumatic separation of the bipartite patella^(49)^. That is probably attributable to the low frequency of the type, which accounts for 5% of reported cases,^(44)^ and an overlap with Sinding-Larsen-Johansson syndrome, because both conditions affect mainly young men who practice physical activity, present knee pain, and have a fragment in the inferior aspect of the patella^(44)^. Probably, the only way to distinguish between the two conditions would be by evaluating previous examinations: a previously divided patella indicating bipartite patella and a previously normal patella indicating Sinding-Larsen-Johansson. There are those who argue that there is no separate ossification center in the lower patella and that cases of supposedly type I bipartite patella are in fact sequelae of Sinding-Larsen-Johansson disease or its asymptomatic form^(46,50)^.
